# Structural Validity and Reliability of a Tool for Clinical Rehabilitation Staff to Evaluate Life-Goal-Setting Practice for Cancer Survivors

**DOI:** 10.3390/curroncol32110625

**Published:** 2025-11-06

**Authors:** Katsuma Ikeuchi, Seiji Nishida, Mari Karikawa, Chiaki Sakamoto, Mutsuhide Tanaka

**Affiliations:** 1 Department of Occupational Therapy, Faculty of Health and Welfare, Prefectural University of Hiroshima, Hiroshima 723-0053, Japan; 2 Department of Nursing, Faculty of Health and Welfare, Prefectural University of Hiroshima, Hiroshima 723-0053, Japan

**Keywords:** cancer rehabilitation, life-goal-setting, clinical rehabilitation staff, quality of life, Rasch analysis, psychometric property

## Abstract

**Simple Summary:**

Cancer survivors often set meaningful life goals with clinical rehabilitation staff, but until now, there has been no valid and reliable tool to evaluate how well staff support this process. This study tested and refined an assessment tool to ensure it accurately measures life goal setting practices. After careful analysis and revisions, the final version contains 14 clear items that clinical rehabilitation staff can use to reflect on their practice. This tool can help identify areas where professionals may need to improve and guide the development of support systems. In the future, it may contribute to improving the quality of rehabilitation services for cancer survivors, inform rehabilitation policies on patient-centered care, and inspire further research into how life goal setting affects recovery and well-being. By strengthening professionals’ ability to support life goal setting, this tool has the potential to improve outcomes for people living with cancer.

**Abstract:**

**Background**: There is a need for an assessment tool for clinical rehabilitation staff to evaluate their life-goal-setting practice, especially in oncology rehabilitation. This study aimed to confirm the structural validity and reliability of the 21-item Reengagement life Goal Assessment Tool for Cancer survivors (ReGAT-C) with a five-category response scale. **Methods**: Participants were clinical rehabilitation staff who worked at designated cancer care hospitals in Japan and had experience in setting life-goals with cancer survivors hospitalized during the non-terminal phase. The ReGAT-C was mailed to participants twice, and Rasch analysis was repeated on the scores of the first ReGAT-C to test structural validity and reliability. The test–retest reliability was also examined using the scores of the first and second ReGAT-Cs after revising it according to the Rasch analysis results. **Results**: A total of 121 participants completed the first ReGAT-C, and 70 participants completed the second ReGAT-C. Following three Rasch analyses, the ReGAT-C was revised to contain 14 items with a three-category response scale. The revised scale showed satisfactory psychometric properties. **Conclusions**: The 14-item ReGAT-C with a three-category response scale could help staff to identify elements that are lacking in their practice and adjust their policies based on the items’ difficulty.

## 1. Introduction

Cancer is associated with various adverse physical, psychological, and social outcomes for many survivors [1,2]. Because global mortality rates from cancer have continuously declined since 1991, resulting in an overall mortality reduction of 33% [3], cancer is now considered a chronic disease. As the chance of surviving cancer increases, the number of survivors experiencing various long-term effects has risen. Therefore, the development of strategies to support the quality of life (QOL) of cancer survivors is increasingly important.

Goal-setting is the formal process by which a rehabilitation professional or team, together with the patient and/or their family, negotiate goals; it should become a central feature in rehabilitation and a core competency of all rehabilitation teams [4]. Research among adults receiving comprehensive rehabilitation, including physical, psychosocial, and vocational rehabilitation interventions for neurological conditions demonstrated that advantages of goal-setting included improvements in health-related QOL, emotional state, and self-efficacy [5]. Another study found that actively involving people with predominantly physical neurological deficits in the goal-setting process resulted in goals being perceived as more relevant, resulting in greater autonomy and satisfaction with goal setting [6].

Life-goals are internal representations of the desired states that motivate behavior; they give meaning to a person’s life and are an important part of identity development [7]. Examples of life-goals include maintaining a healthy lifestyle, spending time with family, traveling, returning to work, and enjoying leisure activities. Although cancer and its treatment may have long-term negative effects on survivors’ life-goals [7], the use of more goal-adjusted strategies, greater progress in achieving goals, and downgrading the importance of unattainable goals can have positive effects on QOL [8,9]. Therefore, it is very important for clinical rehabilitation staff to support cancer survivors in setting life-goals and in managing these goals to improve their QOL.

In response to the lack of tools to assess and promote client-centered goal-setting in healthcare in general, and the importance of goal-setting as a core skill for clinical rehabilitation staff, the Client-Centredness of Goal Setting (C-COGS) scale was developed to evaluate goal-setting in individuals with acquired brain injury [10,11]. The client rates the C-COGS items based on the alignment of rehabilitation goals (e.g., cooking with both hands) with their values and beliefs, their perspective on their involvement in the goal-setting process, and the client-centeredness (e.g., meaningfulness of the individual’s goals and client’s motivation to work toward them) of each goal set. C-COGS scores correlate with self-perceived goal importance and the extent of the therapeutic alliance, which is the relationship between the client and therapist including the feelings or behaviors a client may exhibit in relation to their therapist [10,12].

However, these correlations were derived solely from the clients’ perspectives. While C-COGS provides valuable insight into client-centeredness from one side of the therapeutic relationship in goal-setting process, it is not able to evaluate the equally essential perspective of clinical rehabilitation staff themselves. Therefore, a tool that captures the clinical rehabilitation staff perspective is warranted. Especially in oncology rehabilitation, it may be necessary for both clients and clinical rehabilitation staff to complete tools to assess life-goal-setting. Previous studies have shown that goal-setting practice may generate barriers specific to cancer survivors, such as unknown life expectancy issues and difficulty in understanding the limitations of life-sustaining treatment [13,14]. It has also been reported that clinical rehabilitation staff may be hesitant to attempt patient education about goal-setting, which could explain the low rate of patient education on this issue [15].

To address this issue, we developed the Reengagement life Goal Assessment Tool for Cancer survivors (ReGAT-C). This tool allows clinical rehabilitation staff including physical therapists (PTs), occupational therapists (OTs), and registered nurses (RNs) to evaluate their practice of life-goal-setting for cancer survivors hospitalized during the non-terminal phase. This does not require completion of the tool by cancer survivors, who have been adversely affected both physically and psychologically by cancer diagnoses and the side effects of cancer treatment. Importantly, this could help to minimize the burden on cancer survivors [16].

When developing the ReGAT-C concepts and items, we found that the components of life-goals set by cancer survivors and clinical rehabilitation staff could be grouped as life-goal classification, characteristics (methods of describing goals, including goal content, importance, difficulty, and attainability), and the goal-setting process [16]. The ReGAT-C contains 21 items with a five-category response scale corresponding to these three components (Table 1). The ReGAT-C classifies life-goals into health-related, psychological, social, achievement-related, and leisure goals (Classification A) and learning and performance goals (Classification B). Details of the components and use of the ReGAT-C have been previously published [16].

Although the content validity of the ReGAT-C has been verified [16], its psychometric properties have not yet been confirmed. Therefore, this study aimed to confirm the structural validity and reliability of the ReGAT-C.

## 2. Materials and Methods

### 2.1. Design

This was a cross-sectional survey study using postal questionnaires. The study was designed according to the COnsensus-based Standards for the selection of health Measurement INstruments (COSMIN) study design checklist for patient-reported outcome measurement instruments [17]. Participants were mailed questionnaires twice: the first mailing was to test structural validity and reliability of the ReGAT-C using the Rasch model, and the second mailing was to examine test–retest reliability using classical test theory.

### 2.2. Participant Recruitment and Selection

#### 2.2.1. Eligibility Criteria

The participants were clinical rehabilitation staff (PTs, OTs, RNs) who worked at cancer care hospitals designated by the Japanese Ministry of Health, Labour and Welfare, and who were assigned to cancer survivors who met the following eligibility criteria: (1) over 18 years old; (2) admitted to (or in the process of being admitted to) hospital for cancer treatment; (3) treated or being treated with the expectation of being discharged from hospital; (4) had set life-goals related to life after discharge from the hospital in the context of rehabilitation or nursing practice; (5) at least 1 week had passed since the day the life-goals were set. The reason for eligibility criterion (5) was that the ReGAT-C includes items related to the goal-setting process, so if the goals were set less than 7 days ago, the participant would not be able to answer items 14–21.

Clinical rehabilitation staff who had only worked with cancer survivors who met the following exclusion criteria were not eligible to participate: (1) cancer patients in the terminal stage (individuals whose prognosis for life was expected to be less than 6 months); (2) individuals with dementia, mental illness, or consciousness disorder. Therefore, cancer survivors receiving supportive or palliative treatment without curative intent, but who were not in the terminal stage, were also included as eligible individuals.

#### 2.2.2. Sample Size and Recruitment

The COSMIN group [17] specifies a sample size of 100–199 as adequate when Rasch analysis is used to confirm structural validity, and specifies a sample size of 50–99 as adequate when confirming test–retest reliability. As shown in Table 2, the cancer care hospitals in Japan are categorized into 11 regional divisions (e.g., Hokkaido and Tohoku), with each region accounting for a different proportion of the total. Stratified random sampling based on the regional divisions was used during the recruitment process. We selected 120 departments (60 rehabilitation departments and 60 nursing departments) in 456 hospitals, with reference to a study by Ikechi et al. in which 33.1% of facilities initially agreed to participate and 84.5% of responses obtained were valid [18]. In this study, it was predicted that three participants would participate from each of the institutions that initially agreed to cooperate.

### 2.3. Data Collection

#### 2.3.1. Survey Items

Data on the basic characteristics of the participants, including their profession and the number of years they had worked with cancer survivors, were collected. Participants recalled their most recent experience in setting a life-goal with or for a cancer survivor who met the eligibility criteria, and completed the ReGAT-C. Comments were received concerning difficulty imagining the conditions under which it is recommended to set learning goals rather than performance goals (items 2 and 3) when the content validity was verified [16]. Therefore, participants watched an online educational video explaining the learning and performance goals (Classification B) and the conditions under which these goals should be set before responding to these items.

#### 2.3.2. Processing of Missing Values

If the percentage of missing ReGAT-C data was 5% or less of the total responses, the mode of the scores on the completed items was used to replace the missing value using the imputation method [19,20]. We planned to exclude the data of participants with missing values if the percentage of missing data was 5% or more.

#### 2.3.3. Procedures

The study procedure was divided into three stages. In phase 1, study invitation letters were sent by mail to 120 heads of rehabilitation and nursing departments. Upon agreeing to participate in the study, these individuals were asked to deliver the enclosed leaflets requesting study participation to clinical rehabilitation staff. Those staff who agreed to participate were able to access a web form using a QR code included on the leaflet, and were required to provide their name, affiliation, and mailing address.

In phase 2, participants were sent documents containing the above-mentioned survey items by post, and were asked to reply within 3 weeks. In phase 3, participants who had completed the first postal questionnaire were sent a second ReGAT-C, and asked to complete it within 2 weeks.

### 2.4. Rasch Analysis

To confirm the structural validity and reliability, Rasch analysis was conducted on scores from the 21-item ReGAT-C with a five-category response scale completed by clinical rehabilitation staff in the first postal questionnaire using Winsteps software (version 5.8.0). The principle of the Rasch model is that when a person of a certain ability encounters an item of a certain difficulty, the probability of that person correctly answering the item depends on the difference between the person’s ability and the item’s difficulty [21]. As this type of analysis has the advantage of reducing the number of items on an assessment scale [22], it is increasingly used as a scale development technique in rehabilitation and nursing sciences to produce scales with superior structural validity and reliability [23,24]. The ReGAT-C items that showed abnormal values in the following evaluations were flagged, and their exclusion was considered. Rasch analysis was repeated until the standard ranges were met.

#### 2.4.1. Category Response Scales

We examined whether the ReGAT-C category response scales fit the Rasch model. The judgment criteria were: (1) the observation frequency of each response category was regular, (2) the outfit mean square value (MnSq) was <2.00, and (3) the average measures between each category on the response scale was 1.40–5.00 logits and increased evenly [25]. If these criteria were not met, the category response scale was collapsed and reanalyzed. For example, if response category 1 did not meet the criteria for the five-category response scale, response categories 1 and 2 were collapsed and reanalyzed in a four-category response scale. Furthermore, the following analysis was conducted using the ReGAT-C with the category response scale collapsed.

#### 2.4.2. Targeting

The position relationship between respondent ability and item difficulty was visually confirmed using the Wright map, which visualizes the logit values on a straight line; these values are estimates of the respondent’s ability and the item’s difficulty. In the Rasch model, a respondent with an estimated ability value of 0.00 logit has a 50% probability of success (or failure) on an item with an equivalent estimated difficulty value. Therefore, it is optimal for items to be positioned near the estimated respondent ability values [26].

#### 2.4.3. Item Statistics

The infit and outfit MnSq indicate whether the observed values fit the Rasch estimated values. The infit statistic is an information-weighted indicator of misfit, and it gives relatively more weight to the performance of respondents who are close to the item’s difficulty value [27]. The outfit statistic is not weighted; thus, it remains relatively more sensitive to the influence of outlying scores: the performance of respondents distant from the item’s location. The average MnSq for infit and outfit is 1.00, and the general acceptable range for a rating scale (survey) is 0.60–1.40 [28]. If the average MnSq exceeds 1.40 (i.e., “underfitting”), this may indicate that there are too many unexplained variables in the data. Conversely, if this value falls below 0.60 (i.e., “overfitting”), it is possible that the data are overly fitted to the model, and the data form a too-ideal Guttmann pattern [29].

Winsteps also calculates the standardized Z-score (Zstd) as a fit statistic. The mean value of Zstd is 0.00, the standard deviation (SD) is 1.00, and the acceptable range is ±2.00. If the mean value exceeds +2.00, this suggests underfitting; if it falls below −2.00, this suggests overfitting. However, if the MnSq is within the acceptable range, the Zstd value is not very important and can generally be ignored [29].

#### 2.4.4. Unidimensionality

Principal component analysis (PCA) of residuals was conducted to confirm the unidimensionality of the scale, which is an assumption of the Rasch model. The fit standard was defined as a first contrast eigenvalue for variables that cannot be explained by the Rasch model of <2.00. If the standard value was exceeded, one of the two items with absolute value of standardized residual correlation coefficients of ≥0.40 was removed [30] according to the item’s difficulty, as indicated on the Wright map.

#### 2.4.5. Differential Item Function (DIF)

All respondents with similar abilities should have a similar probability of endorsing items on a scale. Participants were divided into two subgroups according to their profession (therapists and RNs), and the logit values for each subgroup were plotted on a graph to visually assess the DIF. A problematic DIF for an item was defined as a difference of ≥1.00 logit between therapists and RNs [31].

#### 2.4.6. Reliability Based on the Rasch Model

There are two reliability indexes specific to Rasch analysis: person reliability and item reliability. The person reliability index indicates the replicability of person ordering that can be expected if samples of persons were given another parallel set of items measuring the same construct [32]. The item reliability index indicates the replicability of item placement along the pathway if these same items were given to another same-sized sample of persons who behaved in the same way. In Rasch analysis, the indexes corresponding to item reliability and person reliability are the respective separation index and reliability coefficient: person separation reliability index, person reliability coefficient, item separation reliability index, and item reliability coefficient. Positive person reliability is indicated by a person separation reliability index ≥ 2.00 and person reliability coefficient ≥ 0.80, and positive item reliability is shown by an item separation reliability index ≥ 3.00 and item reliability coefficient ≥ 0.90 [33]. Winsteps provides a Cronbach’s alpha coefficient as an indicator of internal consistency; this value should exceed 0.70 to indicate positive internal consistency [34].

### 2.5. Test–Retest Reliability Based on Classical Test Theory

The scores of the first and second ReGAT-C questionnaires were used to confirm the test–retest reliability of the scale. Data for participants who completed the ReGAT-C questionnaire the first time, and then completed the ReGAT-C questionnaire again 10–30 days later, were analyzed. This analysis included only data for participants who did not provide rehabilitation or nursing care to the specific cancer survivors whose data were reported in the first ReGAT-C questionnaire during the two measurement periods. The intraclass correlation coefficient (ICC) (1, 1) was calculated using ReGAT-C data after collapsing the category response scale and deleting items in accordance with the results of the Rasch analysis. As the ICC can be calculated if the data are normally distributed, the raw data were converted to logit values using the Rasch model, and the ICC was then calculated using IBM SPSS Statistics version 29 (Armonk, NY, USA: IBM Corp). ICC (1, 1) values of 0.41–0.60 were considered to indicate moderate reliability, values of 0.61–0.80 substantial reliability, and value of 0.81–1.00 almost perfect reliability [35].

### 2.6. Ethical Considerations

This study was approved by the Research Ethics Committee of the Prefectural University of Hiroshima (approval number: Issue 23MH060). The aims, procedures, voluntary nature of participation, anonymity, and privacy protection were explained using a Participant Information Sheet. All participants then provided their written consent.

## 3. Results

### 3.1. Participants

**Figure 1:**
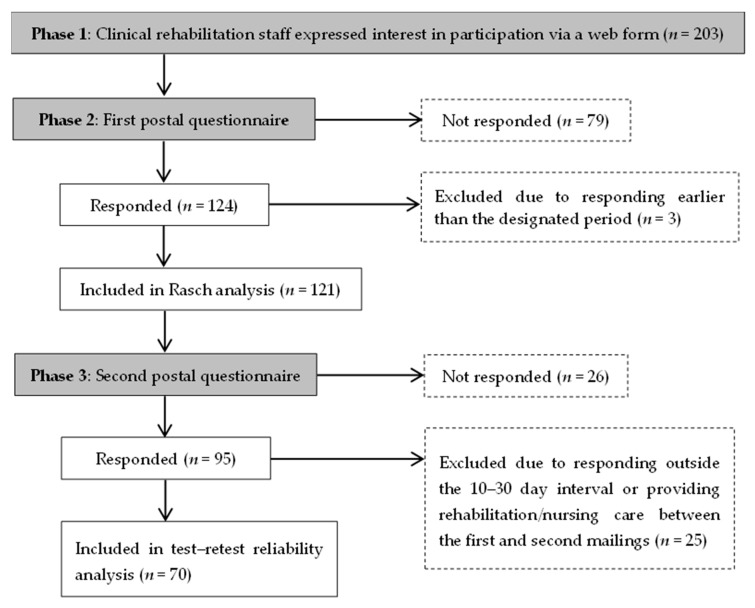
Flow diagram of participant inclusion and analysis.

In phase 1, 203 individuals expressed their intention to participate in the study via the web form, as shown in Figure 1. After the first postal questionnaire was distributed in phase 2, 124 individuals replied (response rate: 61.08%). Of these, the replies of three individuals who completed the ReGAT-C questionnaire within 1 week of the goal-setting date were excluded, and the replies of 121 participants from 35 facilities were included in the analysis (valid response rate: 97.58%). The 121 participants comprised 46 PTs, 28 OTs, and 47 RNs, and the average number of years of experience working with cancer survivors was 9.19 (SD = 6.42). Table 2 shows their regional divisions, with fewer participants from the Kinki and Kyushu regions and more from the Chugoku and Southern Kanto regions.

Examples of the life-goals participants set with cancer survivors included increasing their physical strength, remembering to take their medication, consulting with medical staff about their anxiety, returning to work as a hairdresser, and playing golf as a leisure activity. There were some missing values in the first ReGAT-C, as 4 of the 121 respondents did not answer one item, respectively (0.16% of all responses). Therefore, the mode of the scores on the completed items was imputed for the missing values.

The characteristics of cancer survivors as reported by participants are shown in Table 3. Many of the cancer survivors were men (*n* = 77) and aged ≥ 40 years (*n* = 114). The most common cancer types were digestive (*n* = 30), lung (*n* = 19), hematopoietic (*n* = 16), and urological (*n* = 14). The most common treatments were chemotherapy (*n* = 81) and surgery (*n* = 73). The most common cancer stages were stage IV (*n* = 40) and stage II (*n* = 33). The most common Eastern Cooperative Oncology Group Performance Status (ECOG PS) was 1 (*n* = 49).

### 3.2. First Rasch Analysis

#### 3.2.1. Category Response Scales

In the analysis of the data for the original ReGAT-C (i.e., 21-item, five-category response scale), the frequency of observation of response category 1 was 7.04%, which was lower than that of the other response categories on the scale (Table 4). The outfit MnSq met the standard ranges in all response categories on the scale. The average measures between the response categories were: 1.15 logit between 1 and 2, 0.72 logit between 2 and 3, 0.90 logit between 3 and 4, and 1.74 logit between 4 and 5. Thus, the logit values were less than 1.40 between 1 and 2, 2 and 3, and 3 and 4. Furthermore, there was no equal increase between each response category on the scale.

Based on these results, we considered that response categories 1 to 3 should be collapsed. When these response categories were collapsed and the analysis was repeated with 1 (strongly disagree–neither agree nor disagree), 2 (agree a little), and 3 (strongly agree), the average measure increased evenly by 1.98 logit (Table 4). Therefore, the ReGAT-C with a three-category response scale was used in the following analyses.

#### 3.2.2. Targeting

**Figure 2:**
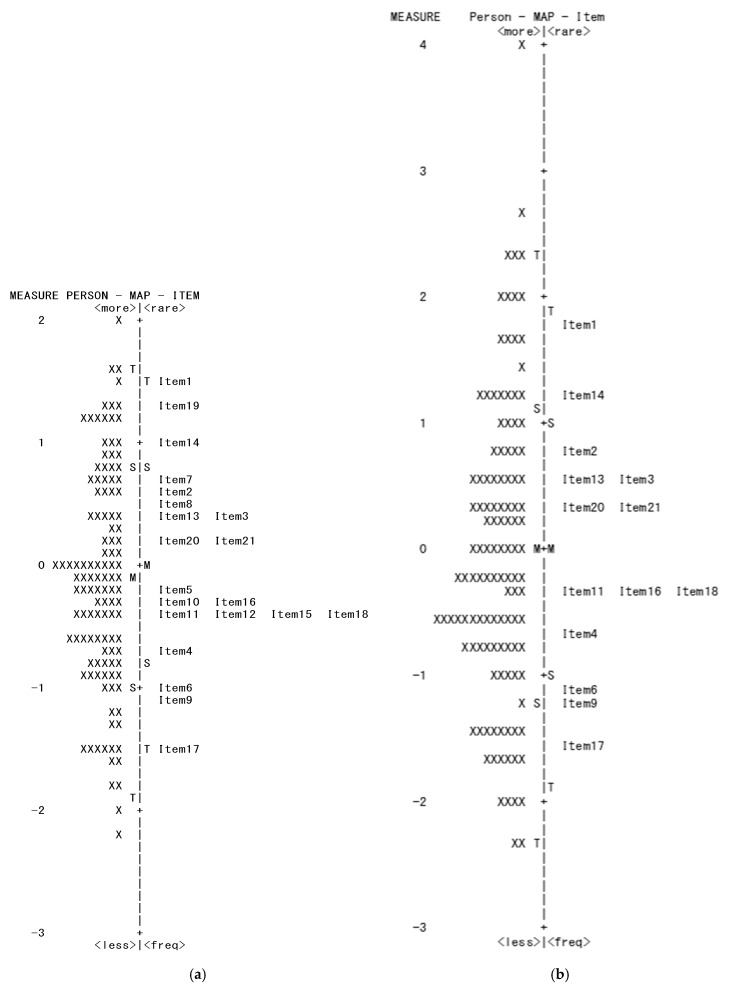
Wright map. The diagram (**a**) shows a Wright map output from the first Rasch analysis. The diagram (**b**) shows a Wright map output from the third Rasch analysis. The M in the center of the Wright map indicates the mean, S indicates 1 standard deviation (SD), and T indicates 2 SD.

The Wright map output showed that the item difficulty (logit) values were distributed within 2 SD, and that there were no items close to the logit values of respondents with very low ability (Figure 2). There were several items with similar logit values.

#### 3.2.3. Item Statistics

The infit MnSq for all items was in the range 0.56–1.84 (Table 5). Item 7 (infit MnSq = 1.84), item 8 (infit MnSq = 1.47), and item 19 (infit MnSq = 1.56) showed underfitting, whereas item 15 (infit MnSq = 0.57) and item 17 (infit MnSq = 0.56) showed overfitting. The outfit MnSq was in the range of 0.56–1.95. Item 7 (outfit MnSq = 1.95), item 8 (outfit MnSq = 1.50), and item 19 (outfit MnSq = 1.47) showed underfitting, whereas item 15 (outfit MnSq = 0.58) and item 17 (outfit MnSq = 0.56) showed overfitting. The Zstd values for item 6 (infit Zstd = −3.26, outfit Zstd = −2.82), item 9 (infit Zstd = −2.44), and item 16 (infit Zstd = −2.87, outfit Zstd = −2.93) showed overfitting.

#### 3.2.4. Unidimensionality

The results of the PCA of the residuals showed that the first contrast eigenvalue of the variables that could not be explained by the Rasch model was 2.67, indicating the existence of a second contrast. The standardized residual correlations were 0.54 between items 4 and 5, and 0.48 between items 15 and 16.

#### 3.2.5. DIF

**Figure 3:**
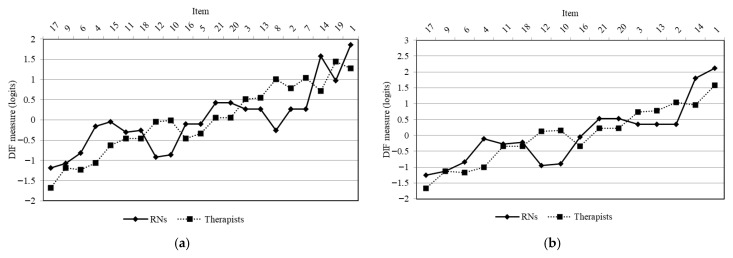
DIF by profession (therapists and nurses). The diagram (**a**) shows the results of the first Rasch analysis. The diagram (**b**) shows the results of the second Rasch analysis. The items on the *x*-axis are arranged in order of decreasing item difficulty (logit value) from left to right. The *y*-axis indicates the logit value. DIF: differential item function; RN: registered nurse.

The difference in logit values between the therapists and RNs on item 8 was 1.25 logit, indicating that the logit value for the therapists was higher and that a DIF was present (Figure 3).

#### 3.2.6. Reliability According to the Rasch Model

The person separation reliability index was 2.10, the person reliability coefficient was 0.81, the item separation reliability index was 4.75, the item reliability coefficient was 0.96, and Cronbach’s alpha coefficient was 0.84.

#### 3.2.7. Identifying Items to Delete

Items that needed deleting were identified using the results of the initial Rasch analysis. As the Zstd value can generally be ignored [29], and the person separation reliability index and person reliability coefficient were close to the boundaries of the standard range, the Zstd value alone was not used to determine which items to delete. The deleted items and the reasons for deletion are shown in Table 6.

Items 7 and 19 were deleted because of underfitting. Item 8 was deleted because of underfitting and DIF. Items 4 and 5, and items 15 and 16, had standardized residual correlations of ≥0.40. After comparing items 4 and 5, item 5 was deleted because there were some items with similar logit values on the Wright map (Figure 2), and the scoping review [15] that was the basis for item generation showed that item 4 was a core characteristic for cancer survivors. After comparing items 15 and 16, item 15 was deleted because it had more items with similar logit values, and it showed overfitting. However, although there was a minor concern about overfitting for item 17, indicating that the item does not add new information different from other items, this item was not deleted. As the easiest item to answer, the overfitting of the data to the Rasch model was considered acceptable.

### 3.3. Second Rasch Analysis

After deleting items 5, 7, 8, 15, and 19, the second Rasch analysis was conducted using the 16-item ReGAT-C (Table 6). The item statistics results showed that only the infit MnSq of item 17 was slightly below the standard range (Table 5). The results of the PCA of residuals showed that the first contrast eigenvalue of the variables that could not be explained by the Rasch model was 2.10, indicating the existence of a second contrast. Although no items met the deletion criterion of absolute value of standardized residual correlations ≥0.40, items 12 and 20 demonstrated a high negative standardized residual correlation (−0.38). The difference between the therapist and RN subgroups for items 10 and 12 was 1.05 and 1.07 logit values, respectively, indicating that the logit value for therapists was higher, and that there was a DIF (Figure 3). The person separation reliability index was 2.07, the person reliability coefficient was 0.81, the item separation reliability index was 5.13, the item reliability coefficient was 0.96, and Cronbach’s alpha coefficient was 0.84.

Regarding the slight concern about overfitting for item 17, as this was the easiest item to answer and it was considered acceptable for the overfitting of the data to the Rasch model, item 17 was not deleted. Therefore, items 10 and 12 were deleted based on the DIF.

### 3.4. Third Rasch Analysis

The third Rasch analysis was conducted using the 14-item ReGAT-C (Table 6). Although the Wright map showed that there were no items close to 0.00 and −2.00 logit, the item difficulty was widely spread (Figure 2). The infit MnSq and outfit MnSq were in the range 0.63–1.36, and all items, including item 17, were within the standard range (Table 5). The results of the PCA of residuals showed that the first contrast eigenvalue of the variable that could not be explained by the Rasch model was 1.95, indicating unidimensionality. This indicated that the deleted item 12 had high negative standardized residual correlation with item 20, and both items comprised the second contrast related to the “assessment of life-goal achievement.”

None of the items showed a DIF exceeding 1.00 logit between the therapist and RN subgroups. The person separation reliability index was 2.03, the person reliability coefficient was 0.80, the item separation reliability index was 5.60, the item reliability coefficient was 0.97, and Cronbach’s alpha coefficient was 0.82.

All the standard ranges were met, and the Rasch analysis was completed. After the third Rasch analysis, there were 14 remaining items, of which 3 were in the goal classification, 5 in the characteristic, and 6 in the process (Table 6).

### 3.5. Test–Retest Reliability

The second ReGAT-C questionnaire was completed by 95 participants (response rate: 78.51%), and there were no missing values (Figure 1). The ICC (1, 1) was calculated using data from participants who did not provide rehabilitation or nursing care to the target cancer survivors during the 10–30-day period in which the first and second ReGAT-C questionnaires were completed. A total of 70 participants (25 PTs, 22 OTs, and 23 RNs) completed the second ReGAT-C on average 16.3 days (SD = 4.2) after the first. The ICC (1, 1) between the total test–retest scores was 0.81 (95% confidence interval, 0.71–0.88), showing substantial to almost perfect reliability.

## 4. Discussion

This study aimed to confirm the structural validity and reliability of the ReGAT-C, which consists of 21 items with a five-category response scale. The results showed that the revised ReGAT-C, which comprises 14 items with a three-category response scale, has satisfactory psychometric properties, also has the important advantage of using an interval scale rather than an ordinal scale as used in the original ReGAT-C. The scale uses self-ratings by clinical rehabilitation staff including PTs, OTs, and RNs caring for non-terminal hospitalized cancer survivors and offers a suitable measure of life-goal-setting practices.

The use of Rasch analysis, which can be used to develop more convenient scales and is useful for reducing scale items [22], was successful in this study. There was a risk that the ReGAT-C components (i.e., the classification, characteristics, and processes of life-goal-setting) would change. However, the 14-item ReGAT-C includes at least three items corresponding to each of the three components, indicating the face validity of this scale.

This study found that the ReGAT-C scale fitted the Rasch model better with a three-category response scale than with a five-category response scale, which may sometimes be inappropriate during Rasch analysis (e.g., when the frequency data for the category response scale are limited, or when it is difficult for the user to differentiate between response categories) [36]. In the ReGAT-C with a five-category response scale, the percentage of participants who selected response categories 4 (agree a little) and 5 (strongly agree) was high at 67.73%, whereas the percentage of participants who selected response category 1 (strongly disagree) was low at 7.04% (Table 4). Therefore, it is likely that respondents found it relatively easy to express agreement with the items on the 21-item ReGAT-C. Furthermore, the interval between the average measures for response categories 2 (disagree a little) and 3 (neither agree nor disagree) was the smallest at 0.72, suggesting that participants had difficulty differentiating between response categories 2 and 3. Therefore, it was reasonable to collapse response categories 1 to 3 into a single response category; these three collapsed response categories should be named “disagree” in further studies that use the ReGAT-C.

Following the three Rasch analyses, several items were deleted. In the analysis of item statistics, items 7, 8, and 19 were deleted because of underfitting. Underfitting suggests that there may be too many unexplained variables in the data [29]. Item 7, being overly careful about the risk of worsening body functions, applies to only some cancer survivors with severe ECOG PS or cancer stage, and may result in suppression of scores on other items. The scores for items 8 and 19 fluctuate according to the frequency of hospital visits by family members of cancer survivors and the opportunity to write down life-goals, rather than the success of life-goals. Therefore, it is considered that the success of life-goals and these item difficulties did not match the Rasch model.

The items that were deleted because of standardized residual correlations were item 5, which correlated with item 4, and item 15, which correlated with item 16. Item 15 also showed overfitting. It was easy to understand intuitively that these items had standardized residual correlations.

Items 8, 10, and 12 were deleted because of DIF. The difficulty of these items was higher for therapists than for RNs. Items 8 and 10 of the ReGAT-C are designed to ensure that the clinical rehabilitation staff using this tool confirm that the intentions of the key person and other professionals (e.g., physicians) align with the life-goals they have set. This observed difficulty for therapists may stem from the fact that they often have fewer opportunities than RNs to ascertain the intentions of key persons and other professionals.

Additionally, item 12 is designed to ensure that the achievement level of the life-goals can be objectively evaluated. The difference in the difficulty level of this item could be explained by the difference in the characteristics of life-goals set between subgroups. The main roles of RNs are to share information to enable participation in decision-making, emotional and support aspects, transition from being a patient in hospital to a survivor in the community, and cancer care management [37]. The main roles of PTs are to improve the physical and psychological complications associated with cancer and its treatment, as well as the prognosis of cancer survivors, through physical activities [38]. The main role of OTs is to improve overall QOL by promoting engagement in meaningful daily activities [39]. Therefore, it is likely that the difficulty of setting life-goals that can be objectively assessed for achievement differed between therapists and RNs because the characteristics of the life-goals set by these subgroups differed.

The present analysis of the DIF between the therapist and RN subgroups indicated that the 14-item ReGAT-C with the three-category response scale can be used without being affected by attribute differences between subgroups [36]. Furthermore, the 14-item ReGAT-C with the three-category response scale met the standard ranges for the person and item reliability and showed an acceptable Cronbach’s alpha coefficient. This confirmed that the 14-item ReGAT-C is an accurate and stable measure of person-rank reproducibility and item difficulty, even in a group that includes therapists and RNs. Moreover, the test–retest reliability analysis confirmed that 14-item ReGAT-C scores are stable from 10 to 30 days if rehabilitation or nursing services are not provided to cancer survivors after completion of the initial ReGAT-C. Therefore, the 14-item ReGAT-C satisfies the various standards of the Rasch model and test–retest reliability and is a suitable tool that shows structural validity and reliability.

### 4.1. Recommended Situations and Timing for Using the ReGAT-C

Many cancer survivors suffer from physical and psychological side effects because of their diagnosis and treatment for cancer [1,2]. In particular, when rehabilitation is provided during hospitalization, where it is assumed that many cancer survivors are confused, clinical rehabilitation staff may be hesitant to ask cancer survivors to complete patient-reported outcomes. The ReGAT-C is a unique assessment tool that can be completed by clinical rehabilitation staff, meaning it is recommended for use in such situations. Furthermore, it is recommended that clinical rehabilitation staff use the ReGAT-C as a mid-term assessment during hospitalization, rather than just as a final assessment before a survivor is discharged. This is because clinical rehabilitation staff can identify elements that are lacking in their practice of life-goal-setting, and adjust their policies based on the ReGAT-C results [16]. The Rasch analysis of this study revealed the items’ difficulty value. If there are many elements lacking in practice, clinical rehabilitation staff can refer to Table 5 and Figure 2 and select items in order of decreasing difficulty (i.e., easy to incorporate into practice).

### 4.2. Limitations and Future Directions

There were several study limitations. First, the number of participants in each profession was not equal: 46 PTs, 28 OTs, and 47 RNs. There are approximately 1,093,000 RNs, 70,000 PTs, and 24,000 OTs working in Japanese medical institutions [40,41,42]; thus, the present sample contained relatively few OT participants.

Second, although stratified random sampling based on participants’ regional divisions was employed to ensure an equitable distribution, the number of participants from certain areas, particularly the Kyushu region, was low. This may be attributable to the disasters caused by heavy rainfall and earthquakes that occurred in this region during the period when the mail-in survey was conducted.

Third, the sample included many cancer survivors with low ECOG PS scores (i.e., high performance on activities of daily living). This may be because one of the eligibility criteria for cancer survivors was that they were receiving or had received treatment with the expectation of hospital discharge. These limitations constrain the representativeness of our findings. Therefore, further studies could enhance the validity of the ReGAT-C by reanalyzing data from a larger sample including more clinical rehabilitation staff in the Kyushu region, OTs from various regions, and survivors with low activities of daily living to compare the findings with those of the present analysis.

Fourth, the participants in this study were limited to clinical rehabilitation staff (PTs, OTs, and RNs) in Japan, where physicians are responsible for prescribing and supervising rehabilitation, a role clearly distinct from that of rehabilitation staff. Moreover, life-goals and expectations are influenced by cultural values [43]. These factors may limit the applicability of the ReGAT-C to Japanese physicians and to clinical rehabilitation staff outside Japan who care for cancer survivors in different cultural contexts. Future studies should re-examine the content validity and psychometric properties of the ReGAT-C across different professionals and cultural contexts to expand its applicability.

Fifth, because of the retrospective nature of the data collected via postal questionnaires, it remains unclear to what extent cancer survivors actively participated in the goal-setting process versus it being clinical rehabilitation staff-led. Future research should investigate the degree of survivor participation using methods such as direct observation and qualitative interviews.

Sixth, there is a possibility of bias due to socially desirable answers, whereby participants were hesitant to report low scores when completing the ReGAT-C. This might have affected the results in which many participants chose response categories 4 (agree a little) or 5 (strongly agree). Further research could examine this bias by asking the same clinical rehabilitation staff to complete in the ReGAT-C for two or more survivors, asking these survivors to complete another assessment tool (e.g., the C-COGS scale which has a different respondent), and analyzing the relationship between the two tools.

Seventh, this study was unable to examine the concept of response shift, which has been discussed in research on QOL among cancer survivors [44,45]. Response shift refers to changes in a respondent’s internal standards, values, or conceptualization that may lead to score variations over time. Although satisfactory test–retest reliability of the ReGAT-C was confirmed in this study, response shift may have influenced score fluctuations in some participants. Clinical rehabilitation staff might have gained new insights or reevaluated their support behaviors after completing the initial ReGAT-C, which could have altered their standards when answering it again. Therefore, when interpreting test–retest reliability, it is necessary to consider not only the stability of scores but also the potential internal changes and professional development among respondents. Future longitudinal studies are warranted to examine response shift more rigorously.

Despite these limitations, the ReGAT-C is a suitable tool for clinical rehabilitation staff to measure life-goal-setting practice for cancer survivors undergoing inpatient treatment in the non-terminal phase. These clinical rehabilitation staff could use the ReGAT-C as a tool to reflect on their life-goal-setting practices, and identify and improve aspects that may be lacking in their practice [16]. Furthermore, as this study succeeded in converting the ReGAT-C raw scores into an interval scale, it will be possible in the future to analyze the relationship between life-goals and other variables, including the QOL of cancer survivors, both cross-sectionally and longitudinally. Additional such studies are strongly recommended, as this could lead to the development of support strategies to improve the QOL of cancer survivors.

## 5. Conclusions

The 14-item ReGAT-C with a three-category response scale shows structural validity and reliability and is a suitable tool for quantitatively measuring clinical rehabilitation staff’s life-goal-setting practices for cancer survivors. The use of the ReGAT-C may also lead to improvements in the life-goal-setting practice, as clinical rehabilitation staff who complete self-ratings may become more aware of those aspects that are lacking in the life-goal-setting practice. Furthermore, it is expected that future cross-sectional and longitudinal studies on the relationship between the life-goal-setting practice and other variables, including the QOL of cancer survivors, will be useful.

## Figures and Tables

**Table 1:** The 21-item version of ReGAT-C used for Rasch analysis.

Subscales	Items
Classification	Number of Classification A ^a^ included in the life goals. You understand the conditions of Classification B ^b^ when you set life goals. The type of life goals in Classification B and their conditions are consistent.
Characteristic	4. There is at least one life goal that the cancer survivor wants to engage in. 5. There is at least one life goal that the cancer survivor thinks is achievable. * 6. There is at least one life goal that the cancer survivor can hold on to while undergoing cancer treatment. 7. The cancer survivor can act on all life goals without risk of worsening body functions. * 8. Key persons for the cancer survivor agree with the content of all life goals. * 9. There is at least one life goal that you think is achievable. 10. There is at least one medical staff from a profession that differs from yours who agrees with the content of all life goals. * 11. All life goals were set after fully discussing them between the cancer survivor and you. 12. There is at least one life goal for which you can objectively assess the extent to which achievement has improved. * 13. There is at least one life goal that specifically defines when, who, and how.
Process	14. The cancer survivor enjoys the process of engaging in life goals. 15. The cancer survivor feels more positive by engaging in life goals. * 16. The cancer survivor feels either accomplishment or satisfaction by engaging in life goals. 17. You can explain to medical staff from a profession that differs from yours why you have set these life goals. 18. After the life goals were established, you reassessed whether the goals or related interventions needed modifications or additions. 19. You share important life goals with the cancer survivor through either the survivor or you by writing them down on paper, so that they do not forget. * 20. You reflect with the cancer survivor on whether there has been progress toward the achievement of the life goals. 21. The cancer survivor can recall what their life goals were.

ReGAT-C: Reengagement life Goal Assessment Tool for Cancer survivors. * Items with asterisks were deleted after the Rasch analysis. ^a^ Classification A: health-related, psychological, social, achievement-related, and leisure goals. ^b^ Conditions of Classification B: It is recommended for learning goals to be set rather than performance goals when at least one of the following four conditions is met; (i) the cancer survivor does not know how to achieve their goals; (ii) the cancer survivor has a low ability to achieve their goals; (iii) the cancer survivor is more likely to become pessimistic if they fail to achieve their goals, viewing it as a serious failure; (iv) the cancer survivor does not know enough about what activities they can do because their abilities have not improved sufficiently, such as at the start of rehabilitation. Reproduced (with minor modifications) from reference [16].

**Table 2:** Percentage of cancer care hospitals ^a^ by Japanese regional division and percentage of study participants included in the Rasch analysis belonging to each division.

Regional Divisions	%
Hokkaido	4.8 (4.1) ^b^
Tohoku	9.9 (9.1)
Northern Kanto	9.4 (9.9)
Southern Kanto	18.0 (23.1)
Hokuriku	5.3 (5.0)
Tokai	9.9 (8.3)
Kinki	14.9 (9.9)
Chugoku	8.1 (19.8)
Sikoku	4.6 (6.6)
Kyusyu	13.8 (4.1)
Okinawa	1.3 (0.0)

^a^ The term “cancer care hospitals” refers to facilities designated by the Japanese Ministry of Health, Labour and Welfare. ^b^ The numbers in parentheses indicate the percentage of study participants included in the Rasch analysis.

**Table 3:** Characteristics of cancer survivors as reported using the ReGAT-C by clinical rehabilitation staff.

	*N* = 121
**Sex (*n*)**	
Male	77
Female	44
**Age, years (*n*)**	
18–39	7
40–64	35
65–74	44
≥75	35
**Cancer types (*n*)**	
Digestive	30
Lung	19
Hematopoietic	16
Urological	14
Head and neck	10
Head (brain tumor)	9
Breast	9
Gynecological	5
Pancreatic	4
Sarcoma	2
Gallbladder	1
Liver	1
Peritoneal	1
**Cancer treatment (*n*)**	
Chemotherapy	81
Surgery	73
Radiotherapy	39
**Stage (*n*)**	
I	14
II	33
III	29
IV	40
Unknown	5
**ECOG PS scale (*n*)**	
0	22
1	49
2	20
3	24
4	6

ReGAT-C: Reengagement life Goal Assessment Tool for Cancer survivors, ECOG PS: Eastern Cooperative Oncology Group Performance Status.

**Table 4:** Category response scale analysis.

	Number of Observations	%	Outfit MnSq	Average Measure
Five-category response scale				
1 (Strongly disagree) ^a^	179	7.04	1.30	−2.02
2 (Disagree a little) ^a^	240	9.45	1.16	−0.87
3 (Neither agree nor disagree) ^a^	401	15.78	0.74	−0.15
4 (Agree a little) ^a^	1019	40.10	0.91	0.75
5 (Strongly agree) ^a^	702	27.63	0.99	2.49
Total	2541			
Three-category response scale				
1 (Strongly disagree–Neither agree nor disagree) ^a^	820	32.27	1.05	−1.98
2 (Agree a little) ^a^	1019	40.10	0.90	0.00
3 (Strongly agree) ^a^	702	27.63	1.06	1.98
Total	2541			

MnSq: mean square value. ^a^ Only item 1 was classified by the number of items in Classification A (health-related, psychological, social, achievement-related, and leisure goals).

**Table 5:** Item statistics.

Item No.	First Rasch Analysis						Second Rasch Analysis						Third Rasch Analysis					
	Logit	SE	Infit		Outfit		Logit	SE	Infit		Outfit		Logit	SE	Infit		Outfit	
			MnSq	Zstd	MnSq	Zstd			MnSq	Zstd	MnSq	Zstd			MnSq	Zstd	MnSq	Zstd
Item 1	1.46	0.17	1.20	1.43	1.14	0.82	1.74	0.18	1.31	2.12 *	1.22	1.22	1.80	0.18	1.35	2.36 *	1.23	1.27
Item 2	0.58	0.15	0.97	−0.24	1.03	0.32	0.78	0.15	1.00	0.06	1.05	0.44	0.78	0.16	1.04	0.38	1.08	0.68
Item 3	0.42	0.14	0.86	−1.28	0.91	−0.76	0.59	0.15	0.90	−0.92	0.92	−0.63	0.58	0.16	0.97	−0.21	1.00	0.07
Item 4	−0.68	0.14	1.06	0.56	1.10	0.84	−0.63	0.15	1.18	1.54	1.23	1.75	−0.71	0.16	1.18	1.54	1.20	1.48
Item 5	−0.24	0.14	0.98	−0.18	1.02	0.24	―						―					
Item 6	−1.05	0.15	0.67	−3.26 *	0.67	−2.82 *	−1.03	0.16	0.74	−2.43 *	0.73	−2.11 *	−1.13	0.16	0.75	−2.25 *	0.75	−1.77
Item 7	0.74	0.15	1.84 *	5.98 *	1.95 *	5.92 *	―						―					
Item 8	0.50	0.14	1.47 *	3.79 *	1.50 *	3.69 *	―						―					
Item 9	−1.14	0.15	0.74	−2.44 *	0.89	−0.81	−1.13	0.16	0.84	−1.38	1.05	0.39	−1.23	0.16	0.93	−0.57	1.23	1.44
Item 10	−0.34	0.14	1.09	0.85	1.07	0.67	−0.25	0.15	1.26	2.22 *	1.32	2.51 *	―					
Item 11	−0.40	0.14	0.95	−0.42	0.97	−0.21	−0.31	0.15	1.03	0.34	1.05	0.47	−0.38	0.15	1.09	0.85	1.10	0.81
Item 12	−0.38	0.14	1.14	1.28	1.17	1.48	−0.29	0.15	1.24	2.10 *	1.27	2.16 *	―					
Item 13	0.44	0.14	1.19	1.67	1.14	1.21	0.61	0.15	1.26	2.18 *	1.20	1.61	0.61	0.16	1.36	2.85 *	1.29	2.19 *
Item 14	0.99	0.15	0.92	−0.62	0.90	−0.70	1.22	0.16	1.03	0.31	1.01	0.09	1.26	0.17	1.05	0.48	1.00	0.04
Item 15	−0.40	0.14	0.57 *	−4.80 *	0.58 *	−4.42 *	―						―					
Item 16	−0.32	0.14	0.72	−2.87 *	0.71	−2.93 *	−0.23	0.15	0.80	−1.90	0.79	−1.93	−0.28	0.15	0.86	−1.30	0.83	−1.42
Item 17	−1.45	0.16	0.56 *	−4.20 *	0.56 *	−3.38 *	−1.47	0.17	0.58 *	−3.90 *	0.61	−2.64 *	−1.60	0.17	0.63	−3.37 *	0.70	−1.78
Item 18	−0.38	0.14	0.89	−1.01	0.91	−0.84	−0.29	0.15	0.97	−0.21	0.96	−0.30	−0.35	0.15	1.02	0.18	1.00	0.06
Item 19	1.26	0.16	1.56 *	3.81 *	1.47 *	2.70 *	―						―					
Item 20	0.19	0.14	0.83	−1.72	0.86	−1.24	0.34	0.15	0.87	−1.19	0.90	−0.84	0.32	0.15	0.83	−1.57	0.84	−1.39
Item 21	0.19	0.14	0.85	−1.46	0.82	−1.70	0.34	0.15	0.90	−0.93	0.85	−1.27	0.32	0.15	0.91	−0.77	0.87	−1.13

MnSq: mean square value; Zstd: standardized Z-score; SE: standard error. * Values with asterisks are out of the standard range.

**Table 6:** Remaining and deleted items in each Rasch analysis.

	First Rasch Analysis		Second Rasch Analysis		Third Rasch Analysis
	Remaining Items	Deleted Items (Reason)	Remaining Items	Deleted Items (Reason)	Remaining Items
Classification	Items 1–3	None	Items 1–3	None	Items 1–3
Characteristic	Items 4–13	Item 5 (standardized residual correlations) Item 7 (underfitting) Item 8 (underfitting, DIF)	Items 4, 6, 9–13	Item 10 (DIF) Item 12 (DIF)	Items 4, 6, 9, 11, 13
Process	Items 14–21	Item 15 (standardized residual correlations, overfitting) Item 19 (underfitting)	Items 14, 16–18, 20–21	None	Items 14, 16–18, 20–21
Total number of items	21	5	16	2	14

DIF: differential item function.

## Data Availability

The datasets analyzed during the current study are not publicly available owing to reasons of protection of the individual privacy of the participants.

## Abbreviations

The following abbreviations are used in this manuscript:

ReGAT-C	reengagement life goal assessment tool for cancer survivors
QOL	quality of life
C-COGS	client-centredness of goal setting
PT	physical therapist
OT	occupational therapist
RN	registered nurse
COSMIN	consensus-based standards for the selection of health measurement instruments
MnSq	mean square value
Zstd	standardized Z-score
SD	standard deviation
PCA	principal component analysis
DIF	differential item function
ICC	intraclass correlation coefficient
ECOG PS	Eastern Cooperative Oncology Group performance status
